# New Dental Periodical

**Published:** 1852-07

**Authors:** 


*New Dental Periodical.*—Dr, Solyman Brown, of the City of New York, has just issued the first No. of a Semi-Annual Quarto Magazine of twelve pages in double columns, devoted to the cause of Dental Literature and Science, and suited to the wants of the public and the interests of the profession.

The first No. contains the Editor's well known Didactic Poem, "DENTALOGIA," which treats of the "Diseases of the Teeth and their proper Remedies," in five cantos, as originally published by Dr. E. Parmly, for private distribution among his friends.

The second No. will be issued on the 1st November next, and will contain the Editor's Poetical Dissertation on the "Laws of Health and the causes of Longevity," entitled "DENTAL HYGEIA," in consequence of the close connection between all constitutional disorders and the diseases of the teeth.

The third No. will contain the commencement of a series of articles on "MECHANICAL DENTISTRY," in its present improved condition, illustrated with engravings, being a thorough revision of the author's treatise on that subject as originally published in the 2d Vol. 1st Series of the American Journal of Dental Science.

Each No. of this work will be mailed to all orders containing a remittance of 25 cents, post-paid or not.

For one dollar, six successive numbers which will constitute one entire volume, will be sent as ordered. This volume will complete the treatise on Mechanical Dentistry, block-work and plate-work.

The Nos. will be issued more frequently than *twice a year,* if the state of the subscription list should warrant it.

The first and second Nos. of this volume, will be eminently calculated to be useful to the public as well as the profession; consequently, any dentist who shall transmit to the Editor, 333 Broadway, New York, the sum of five dollars, shall receive one hundred copies for distribution; for eight dollars he shall receive two hundred copies; for ten dollars three hundred copies." The well known reputation of the Dr. as a writer, will evidently secure an extensive circulation.—Ed.

